# Genomic and Molecular Signatures of Successful Patient-Derived Xenografts for Oral Cavity Squamous Cell Carcinoma

**DOI:** 10.3389/fonc.2022.792297

**Published:** 2022-04-04

**Authors:** Wei-Chen Yen, Ian Yi-Feng Chang, Kai‐Ping Chang, Chun‐Nan Ouyang, Chiao-Rou Liu, Ting-Lin Tsai, Yi-Cheng Zhang, Chun-I Wang, Ya-Hui Wang, Alice L. Yu, Hsuan Liu, Chih-Ching Wu, Yu-Sun Chang, Jau-Song Yu, Chia-Yu Yang

**Affiliations:** ^1^ Department of Otolaryngology Head and Neck Surgery, Chang Gung Memorial Hospital, Taoyuan, Taiwan; ^2^ Molecular Medicine Research Center, Chang Gung University, Taoyuan, Taiwan; ^3^ Department of Neurosurgery, Chang Gung Memorial Hospital, Taoyuan, Taiwan; ^4^ College of Medicine, Chang Gung University, Taoyuan, Taiwan; ^5^ Graduate Institute of Biomedical Sciences, College of Medicine, Chang Gung University, Taoyuan, Taiwan; ^6^ Department of Medical Biotechnology and Laboratory Sciences, College of Medicine, Chang Gung University, Taoyuan, Taiwan; ^7^ Department of Microbiology and Immunology, College of Medicine, Chang Gung University, Taoyuan, Taiwan; ^8^ Radiation Biology Research Center, Institute for Radiological Research, Chang Gung University/Chang Gung Memorial Hospital, Linkou, Taiwan; ^9^ Institute of Stem Cell and Translational Cancer Research, Chang Gung Memorial Hospital at Linkou, Taoyuan, Taiwan; ^10^ University of California San Diego, San Diego, CA, United States; ^11^ Division of Colon and Rectal Surgery, Chang Gung Memorial Hospital, Taoyuan, Taiwan; ^12^ Department of Biochemistry and Molecular Biology, College of Medicine, Chang Gung University, Taoyuan, Taiwan; ^13^ Liver Research Center, Chang Gung Memorial Hospital, Linkou, Taiwan

**Keywords:** patient-derived xenografts, oral cavity squamous cell carcinoma, whole-exome sequencing, transcriptome sequencing, engraftment ability

## Abstract

**Background:**

Oral cavity squamous cell carcinoma (OSCC) is an aggressive malignant tumor with high recurrence and poor prognosis in the advanced stage. Patient-derived xenografts (PDXs) serve as powerful preclinical platforms for drug testing and precision medicine for cancer therapy. We assess which molecular signatures affect tumor engraftment ability and tumor growth rate in OSCC PDXs.

**Methods:**

Treatment-naïve OSCC primary tumors were collected for PDX models establishment. Comprehensive genomic analysis, including whole-exome sequencing and RNA-seq, was performed on case-matched tumors and PDXs. Regulatory genes/pathways were analyzed to clarify which molecular signatures affect tumor engraftment ability and the tumor growth rate in OSCC PDXs.

**Results:**

Perineural invasion was found as an important pathological feature related to engraftment ability. Tumor microenvironment with enriched hypoxia, PI3K-Akt, and epithelial–mesenchymal transition pathways and decreased inflammatory responses had high engraftment ability and tumor growth rates in OSCC PDXs. High matrix metalloproteinase-1 (MMP1) expression was found that have a great graft advantage in xenografts and is associated with pooled disease-free survival in cancer patients.

**Conclusion:**

This study provides a panel with detailed genomic characteristics of OSCC PDXs, enabling preclinical studies on personalized therapy options for oral cancer. MMP1 could serve as a biomarker for predicting successful xenografts in OSCC patients.

## Introduction

Oral cavity squamous cell carcinoma (OSCC) is an aggressive disease globally; the overall 5-year survival rate of patients with advanced stage disease has remained lower than 40% (1). OSCC often occurs in the oral cavity due to many etiological factors. Smoking, areca nut products, and alcohol consumption remain the most common risk factors for OSCC in the world (2). Environmental factors such as irradiation, air pollution, and viral infection may increase the risk of gene mutations (3, 4). The activation of oncogenes (such as EGFR, PIK3CA and AKT) and the inhibition of tumor suppressor genes (such as TP53) promote the tumorigenesis of OSCC (5). Most OSCC tumors in male patients occur at the buccal area and tongue (6). The high mortality of OSCC patients is attributed to a late diagnosis, suggesting that early detection is the most effective strategy to ameliorate the outcome and therapy (7).

Many human tumor models have been generated in immune-deficient mice by the subcutaneous or orthotropic injection of various cancer cell lines established from humans to predict the treatment responses of various cancer therapies, including chemotherapy, targeted therapy, and small molecule inhibitors (8). Cell line-derived xenografts serve as well-known models because they are quickly and easily created, and tumors can be quickly acquired (after approximately two to three weeks). However, cancer cell lines may develop different phenotypes during *in vitro* culture conditions. Cell line-derived xenografts may not entirely resemble their parental tumors. Patient-derived xenograft models (PDXs) have been established as useful tools to retain the genetic signatures of patients’ primary tumors (9). Small pieces of tumors from cancer patients were surgically transplanted into immune-deficient mice, followed by tumor growth and transplantation into a second mouse model. PDXs often maintain the cellular and histopathological structures of the original tumors (10). All of these characteristics demonstrate that PDXs are more useful models that are authentic to the environment of the original patient than cell-line xenografts (11). These models can be used for clinical outcome prediction, preclinical drug evaluation, biomarker identification, biologic studies, and personalized medicine strategies (12).

In the present study, we established a panel of OSCC xenografts from a Taiwanese population and characterized the clinical characteristics, genomic landscapes, and transcriptomic signatures between the primary tumors and their matched PDXs. We performed whole-exome sequencing (WES) and RNA sequencing (RNA-seq) analyses on 12 case-matched tumors and PDXs. Our study demonstrated that the genomic and transcriptomic signatures were conserved in most OSCC PDXs. Furthermore, we identified the impact of some biological pathways that were highly associated with tumor engraftment ability in xenografts. Patients with increased activation of the HIF-1 signaling, PI3K-Akt signaling, or epithelial-mesenchymal transition (EMT) pathway and decreased interferon or IL6 immune responses might facilitate the tumor engraftment ability. Overall, we provide a panel of OSCC PDXs for preclinical drug testing and predictive biomarkers for successful engraftment.

## Materials and Methods

### Patient Characteristics

**Figure 1:**
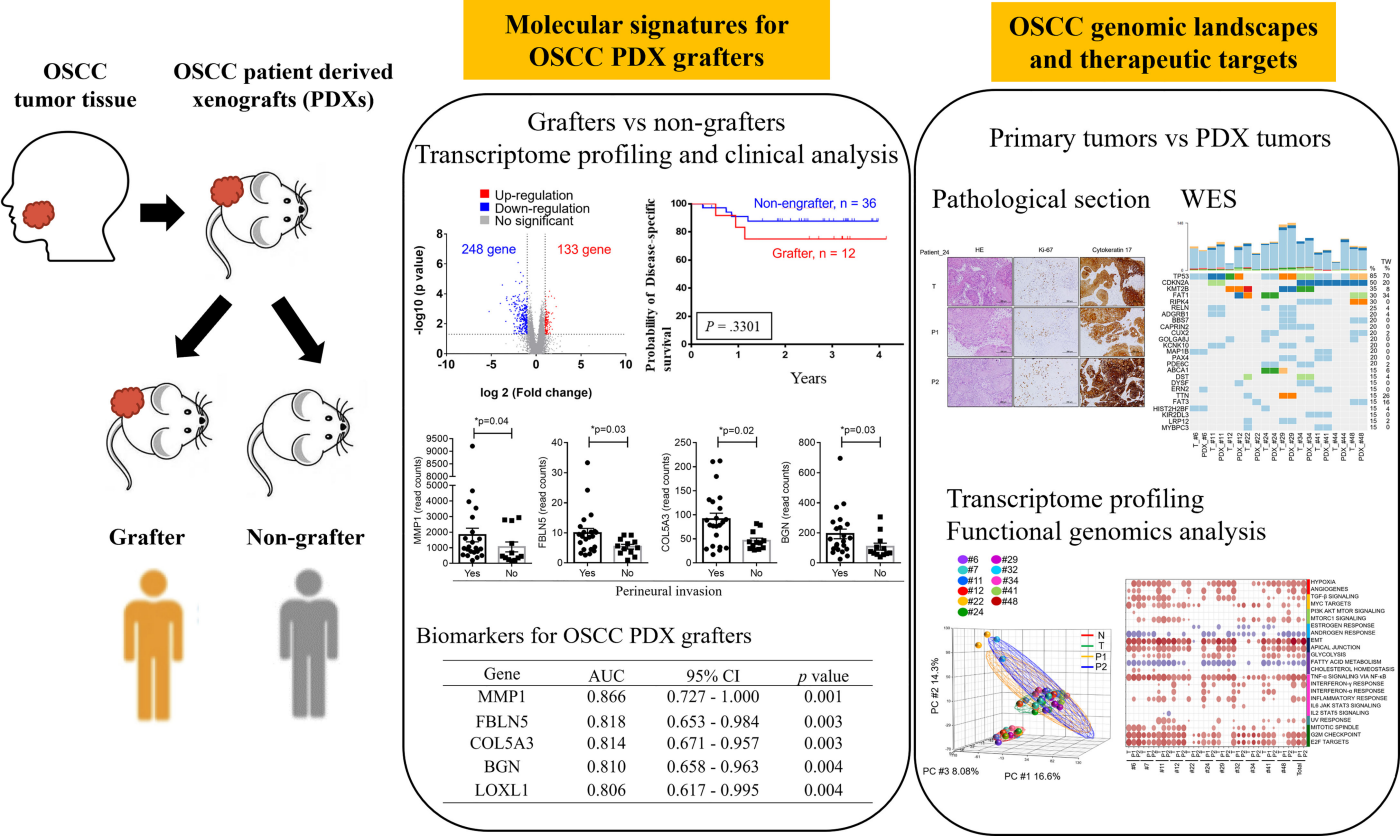
Workflow for the establishment and characterization of OSCC PDX models. Overall, 49 OSCC patients were enrolled in this study. Of the 49 tumors, 12 were successfully engrafted in NSG mice as PDX passage 1 (P1) and PDX passage 2 (P2). The specimens were subjected to pathology analysis, whole-exome sequencing, and RNA-seq. Finally, genomic landscape analysis and gene expression pathway annotation were performed to characterize the molecular signatures in patients and their matched xenografts.

Treatment-naïve OSCC patients were enrolled at Chang Gung Memorial Hospital, Taiwan. This study was approved by the Institutional Review Board at Chang Gung Memorial Hospital, Taiwan (Protocol Nos.: 201800700B0, 102-5685A3). Prior to sample collection, written informed consent was obtained from all participants. Patients underwent clinical examinations, including a physical examination, computed tomography or magnetic resonance imaging of the head and neck, chest radiography, a bone scan and an abdominal ultrasound, according to standard procedures. Primary tumors were excised and transplanted into immune-deficient mice. The demographics, clinical characteristics, and histopathological features of the patients (N = 49) are shown in **Table 1** and **Supplementary Table 1**. All patients had regular follow-ups every 2 months for the first year, every 3 months for the second year, and every 6 months thereafter. Twelve OSCC PDXs were successfully established, and the clinicopathological characteristics were shown in **Table 2**. Primary tumors, adjacent normal tissues, and xenograft tumors were subjected to pathology, WES, transcriptome sequencing, and pathway analyses (**Figure 1**).

**Table 1:** Clinical characteristics of total patients (n = 49) in this study.

Patient categories	Case number n = 49	Engrafter		*p* value
		Yesn = 12	Non = 37	
**Age (years)** ^a^	52 ± 10	51 ± 8	53 ± 10	0.667
**Sex**				
Male	45	11	34	1.000
Female	4	1	3	
**Tumor classification**				
T1 - T2	19	4	15	0.743
T3 - T4	30	8	22	
**Node classification**				
N = 0	18	4	14	1.000
N > 0	31	8	23	
**Overall TNM stage**				
I - II	5	2	3	0.584
III - IV	44	10	34	
**Extranodal extension**				
No	27	5	22	0.331
Yes	22	7	15	
**Perineural invasion**				
No	18	1	17	0.035^b^
Yes	31	11	20	
**Tumor depth (mm)****^a^**	21 ± 16	22 ± 14	21 ± 16	0.925

a These data are presented as mean ± standard deviation.

b This is considered statistically significant.

**Table 2:** The clinicopathological characteristics of 12 OSCC PDX grafters.

Patient number	Age (years)	Gender	T stage	N stage	Pathology	Overall stage	Alcohol drinking	Betel quid chewing	Cigarette smoking	Site
**6**	54	M	2	0	Well	II	Y	N	N	Buccal mucosa
**7**	45	M	1	2B	Poorly	IV	Y	Y	Y	Mouth floor
**11**	48	F	4A	2C	Moderately	IV	N	N	N	Tongue
**12**	44	M	4A	2C	Poorly	IV	Y	Y	Y	Tongue
**22**	56	M	4A	0	Well	IV	Y	Y	Y	Buccal mucosa
**24**	60	M	2	2B	Moderately	IV	Y	Y	Y	Buccal mucosa
**29**	66	M	4A	0	Moderately	IV	Y	Y	Y	Others
**32**	60	M	2	0	Moderately	II	Y	Y	Y	Buccal mucosa
**34**	47	M	4A	2B	Moderately	IV	Y	Y	N	Tongue
**41**	41	M	4A	2B	Moderately	IV	Y	Y	Y	Others
**44**	40	M	2	0	Moderately	II	N	Y	Y	Buccal mucosa
**48**	49	M	3	1	Moderately	IV	N	Y	Y	Others

Y, Yes; N, No; M, male; F, female.

### PDX Model Establishment

NOD.Cg-Prkdcscid Il2rgtm1Wjl/SzJ (NSG) mice (obtained from The Jackson Laboratory) were used in this study and housed in a specific-pathogen-free animal room. All animal experiments were conducted in accordance with the Institutional Animal Care and Use Committee of Chang Gung University (Protocol Nos.: CGU106-114 and CGU107-074). For PDX establishment, tumors from the surgical specimens of OSCC patients were engrafted into NSG mice. In brief, fresh tumor tissues were first washed with PBS containing antibiotic-antimycotic solution (Gibco, USA) and then cut into small pieces of approximately 1 mm^3^. To establish the first-generation (P1) PDX, tumor fragments weighing 50–100 mg were subcutaneously inoculated into the left flank of NSG mice. Tumors reaching approximately 1000 to 1500 mm^3^ were harvested and passaged into another mouse to establish the next generation (P2).

### Histological Characterization

Tumor tissues were formalin fixed and paraffin embedded (FFPE), and tissue sections were stained with hematoxylin and eosin (H&E). Tissue sections (5 μm thick) were subjected to antigen retrieval using Bond Epitope Retrieval Solution 2 in a Bond-Max automated immunostainer (Leica Biosystems) and stained with antibodies against cytokeratin 17 (Cell Signaling Technology) and Ki-67 (Cell Signaling Technology). The procedures were performed in accordance with standard protocols.

### Whole Exome Sequencing (WES)

Genomic DNA was extracted from paired adjacent normal tissues, tumor tissues, and xenograft tissues using the QIAamp DNA Mini Kit (Qiagen), and high-quality genomic DNA was captured using a SureSelect Human All Exon V6 + COSMIC Kit (Agilent Technologies) for exome-captured libraries. The libraries were sequenced using a HiSeq 2000 with the TruSeq PE Cluster kit v3 and TruSeq SBS kit v3 (all from Illumina) according to the manufacturer’s protocol.

### PDX FASTQ Cleaning

To remove mouse genomic DNA from the PDX WES or RNA-seq paired-end reads, we first used Trimmomatic (version 0.38) (13) to remove sequencing adapters and low-quality bases (“ILLUMINACLIP:/trimmomatic-0.38/adapters/TruSeq3-PE.fa:2:30:10” SLIDINGWINDOW:4:5 LEADING:5 TRAILING:5 MINLEN:25). Second, BWA-mem (0.7.15) (for WES data) (https://arxiv.org/abs/1303.3997) or STAR 2.7.3a (14) (for RNA-seq) was used to align the trimmed paired-end reads to the GENCODE V32 human hg38 genome (https://www.gencodegenes.org/human/release_32.html) and to the GENCODE M22 mouse genome (https://www.gencodegenes.org/mouse/release_M22.html). Third, we sorted the human and mouse BAM files by read names with the SAMtools (version 1.9) sort module. Finally, we used Disambiguate (15) and the GATK SamToFastq module to extract human-specific aligned reads from the sorted BAM files.

### WES Data Analysis

We processed the human-specific WES reads into analysis-ready BAM files following the data preprocessing workflow with GATK version 4.1.4.1 (https://gatk.broadinstitute.org/hc/en-us/articles/360035535912-Data-pre-processing-for-variant-discovery). The sequenced reads were mapped to the hg38 reference genome by BWA-mem (0.7.15). The BWA genome index and known single-nucleotide polymorphisms (SNPs), germline resources, and associated files were downloaded from the Google Cloud bucket of the GATK resource bundle (Grch38/Hg38 Resources, https://gatk.broadinstitute.org/hc/en-us/articles/360035890811-Resource-bundle). Read group information was added to SAM files at the alignment stage. SAM files were converted to BAM files with the GATK MergeSamFiles module. The BAM files were further processed by marking duplicates, sorting by chromosome coordinates, and recalibrated by the base quality score with the GATK MarkDuplicates, SortSam, BaseRecalibrator, and ApplyBQSR modules. The CollectHSMetrics module of GATK was used to create the coverage report from the analysis-ready BAM files. The somatic variant calling pipeline was adopted from the GATK guidelines (https://gatk.broadinstitute.org/hc/en-us/articles/360035894731-Somatic-short-variant-discovery-SNVs-Indels-). The candidate somatic short variants were identified from the analysis-ready BAM files by the GATK Mutect2 module. Next, we used the GATK GetPileupSummaries and CalculateContamination modules to construct an estimated fraction of reads due to cross-sample contamination. Finally, we used GATK FilterMutectCalls to identify somatic single-nucleotide variants (SNVs) and indel mutations. The identified SNVs/indels were annotated by Annovar (16) with GENCODE V32 annotation. The R package CopywriteR (version 2.18.0) (17) with default parameters was used to analyze the somatic copy number alterations (SCNAs) from the analysis-ready BAM files. Next, we used GISTIC2 (version 2.0.23) (18) to identify significant SCNA regions from the paired WES BAM files (with parameters -ta=0.4, -td=0.4, and -conf=0.99). Because we used the hg38 reference genome for WES data processing, in GISTIC2, we downloaded the hg38 version of the reference file hg38.UCSC.add_miR.160920.refgene.mat from the Broad Institute FTP site (ftp://ftp.broadinstitute.org/pub/GISTIC2.0/refgenes/). GISTIC2 reported arm- and focal-level SCNAs for the cohort with the G-Score and false discovery rate (FDR) Q value. Only genes located in the focal region with a GISTIC2 Q value less than 0.25 were used for further analysis.

### RNA-Seq, Gene Expression and Pathway Analyses

RNA-seq and data analyses were performed according to our previous reports (19). Briefly, total RNA from paired adjacent normal tissues, tumor tissues, and xenograft tissues was extracted using TRIzol Reagent (Gibco BRL). For RNA-seq, 2 μg purified total RNA was enriched by poly-A tail beads, fragmented and then reverse transcribed into cDNA, and libraries were prepared using the TruSeq Stranded mRNA Sample Preparation Guide (Part # 15031047 Rev. E; Illumina) according to the manufacturer’s instructions. Sequencing was conducted on an Illumina NextSeq 500 instrument. The human-specific RNA reads were mapped to the hg38 reference by STAR (version 2.7.3a). Aligned reads were then normalized and quantified for quantitative representation. Cancer hallmark enrichment analysis was performed according to the gene set enrichment analysis (GSEA)/Molecular Signatures Database (MSigDB; 6.2), and the enrichment score was determined by calculating the probability of overlap between the test set and the hallmark sets using the DoGsea function of the Bioconductor package clusterProfiler (3.12.0). Differentially expressed genes (DEGs, 2-fold difference between groups, p value < 0.05, FDR < 0.05) were selected and subjected to the Database for Annotation, Visualization and Integrated Discovery (DAVID) v6.8 for pathway annotations.

### Statistical Analysis

Patient characteristics were analyzed by the chi-square test, Fisher’s exact test, or the Wilcoxon test. Multivariate models were applied to analyze overall survival and disease-free survival. Survival rates were estimated by Kaplan–Meier plotting and compared by the log-rank test. Statistical analyses were performed using SAS software (v.9.3) or SPSS software (version 20). The significance level was set at p < 0.05.

## Results

### Establishment of OSCC PDX Models

Between 2015 and 2019, we performed a PDX study of OSCC across 49 specimens from treatment-naïve primary patients (**Table 1**). The surgically resected tumor specimens were immediately implanted subcutaneously into NOD/SCID/IL2Rγ null (NSG) mice as passage 1 (P1) generation PDXs; tumors from established P1 PDXs were then transplanted to other NSG mice as passage 2 (P2) generation PDXs and so on (**Figure 1**). Of these 49 specimens, 12 PDXs were successfully established (**Table 2**). There was no significant difference in tumor stage (T), nodal stage (N), overall clinical stage, pathology, or tumor sites between grafters (n = 12) and nongrafters (n = 37) **(** **Table 1** and **Supplementary Table 1**). However, compared with only 54% of nongrafters who were positive for perineural invasion, approximately 91.6% of grafters were positive (p = 0.035; **Table 1**). The overall survival (OS) and disease-specific survival (DSS) differences in grafters versus nongrafters were investigated. The 4-year OS rate was slightly lower for grafters than for nongrafters (p = 0.619) **(** **Supplementary Figure 1**). The 4-year disease-specific survival rate of grafters was also slightly lower (p = 0.33) **(** **Supplementary Figure 1**). Tumors from passage 1 (P1) generation PDXs were harvested to create second generation (P2) xenografts when the tumor size reached approximately 1000 mm^3^ to 1500 mm^3^, with a median time to passage of 84 days (range, 42-105 days). The patients’ primary tumors and their matched P1 and P2 xenografts were further analyzed for pathology, genomic landscapes, and gene expression profiles (**Figure 1**). Histological comparison of PDXs and corresponding primary tumors revealed a high degree of similarity (**Supplementary Figure 2**). Cytokeratin 17 (CK17) is an epithelial marker for squamous cell carcinoma, and its expression is associated with the differentiation and malignancy of OSCC (20). The expression levels of CK17 and the proliferation marker protein Ki-67 were immunohistochemically examined in 12 patients with OSCC and their matched PDX tissues using human-specific anti-CK17 and anti-Ki-67 antibodies, respectively. As shown in **Supplementary Figure 2**, the immunohistochemical staining patterns of CK17 and Ki-67 were similar in most tumors from the two represented patients and their matched PDXs. Pathological examination of the primary tumors and xenografts also confirmed the histopathology of squamous cell carcinoma in our samples.

### Molecular Signatures of Successful Grafters Versus Nongrafters

**Figure 2:**
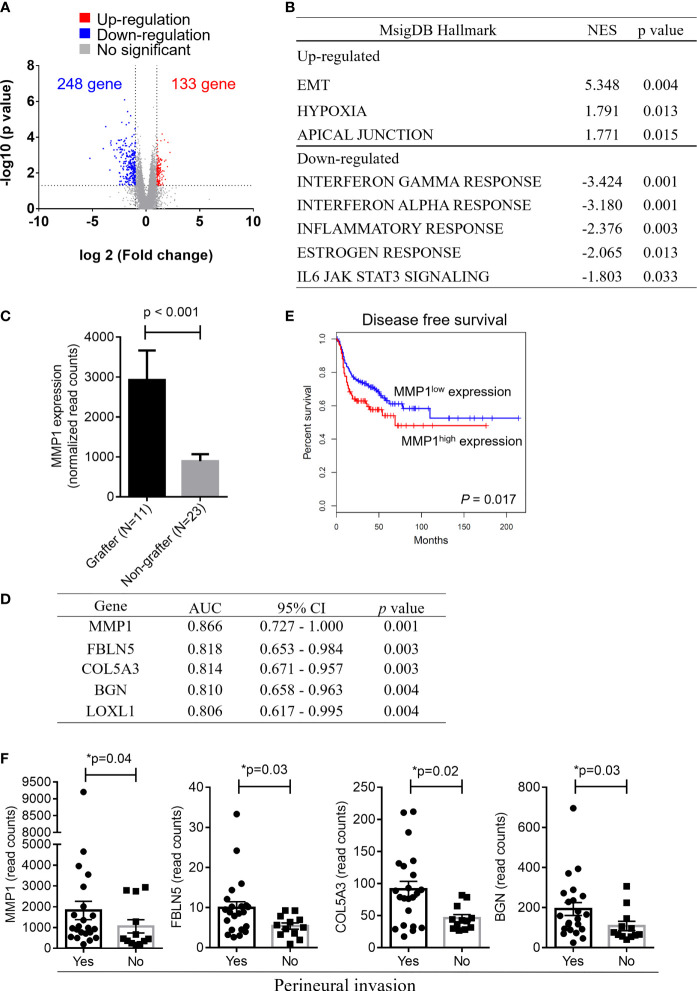
Gene expression signatures in OSCC grafters for PDXs. Among the 49 OSCC patients enrolled in this study, 34 OSCC, including grafters (n = 11) and nongrafters (n = 23), were subjected to RNA-seq analysis. **(A)** The volcano plot displays DEGs from RNA-seq data between the grafter and nonengrafter groups. The x-axis shows the log2-fold change values, and the y-axis shows the -log10 p values for the differentially expressed genes. **(B)** The differentially expressed pathways between nongrafters and grafters were determined by GSEA. **(C)** A bar chart of MMP1 expression between grafters (n=11) and nongrafters (n=23) by RNA-seq. **(D)** An AUC ranking table of the top five genes (MMP1, FBLN5, COL5A3, BGN, and LOXL1) with an AUC higher than 0.8 for distinguishing grafters from nongrafters. **(E)** Kaplan‐Meier plot showing the disease-free survival for patient subgroups stratified by high versus low gene expression of MMP1 among the 514 patients in the HNSCC‐TCGA dataset. The p values were calculated using log‐rank tests. **(F)** The expression of MMP1, FBLN5, COL5A3, and BGN by RNA-seq analysis in oral cancer patients with or without perineural invasion. The p values were calculated using the Mann–Whitney U test. The P value < 0.05 indicated statistical significance (*: p < 0.05).

Although PDX models have emerged as powerful tools for reflecting the original features of patient tumors, the low success rate of PDX establishment is an obstacle that needs to be overcome. Among the 49 OSCC patients enrolled in this study, a total of 34, including grafters (n = 11) and nongrafters (n = 23), underwent RNA-seq analysis to determine the gene signatures that may serve as biomarkers for predicting the successful establishment of PDXs in OSCC patients. The log2 value of the expression fold change and the -log10 value of the p value between nongrafters and grafters were visualized in a volcano plot (**Figure 2A**). The volcano plot revealed 381 significantly altered transcripts (248 (65.1%) downregulated genes and 133 (34.9%) upregulated genes) in the engrafter group. Pathway analysis revealed that the screened genes were significantly associated with several common cancer-associated pathways (**Figure 2B**). The grafter group was significantly enriched in EMT (NES = 5.348, p = 0.004), hypoxia (NES = 1.791, p = 0.013) and apical junctions (NES = 1.771, p = 0.015), and the nonengrafter group was significantly enriched in the interferon gamma response (NES = -3.424, p = 0.001), interferon alpha response (NES = -3.180, p = 0.001), inflammatory response (NES= -2.376, p = 0.003), estrogen response (NES = -2.065, p = 0.013) and IL6 JAK STAT3 signaling (NES = -1.803, p = 0.033). We then selected genes involved in the regulation of EMT biological processes or immune responses and calculated the score based on the geometric mean of their expression levels. The EMT score was also significantly upregulated in grafters compared with nongrafters (**Supplementary Figure 3A**, left panel). However, we observed that the immune response score was slightly increased in nongrafters compared with grafters **(** **Supplementary Figure 3A**, right panel). A heat map of the top 30 dysregulated genes in the EMT or immune response pathway is shown in **Supplementary Figure 3B**. These results revealed that an immunosuppressive microenvironment and activation of the EMT pathway could improve tumor growth in PDX models. Further, the top differentially expressed genes which involved in hypoxia and PI3K signaling pathways in grafters and nongrafters were shown in **Supplementary Figure 3C**.

As shown in **Figure 2C**, the expression of matrix metalloproteinase-1 (MMP-1), which was the most dominant expressed gene in our cohort of grafters, was significantly higher than that in nongrafters. The ability to distinguish between grafter and nonengrafter genes was evaluated by the area under the receiver operating characteristic (ROC) curve (AUC) and ranked in **Figure 2D**. The AUC values of the top five genes (MMP1, FBLN5, COL5A3, BGN and LOXL1) were above 0.800, and their *p* values were less than 0.01. We selected a marker panel with top three differentially expressed genes (MMP1, FBLN5 and COL5A3), which had an AUC value of 0.917 (95% CI = 0.792–1.042) in discriminating grafters from the non-grafters. MMP-1 is a part of the matrix metalloproteinase family that enzymatically degrades the extracellular matrix (ECM) or basement membrane (21). Numerous studies have suggested that MMP1 is associated with tumor invasion and metastasis (22, 23). MMP1 has also been reported as a potential diagnostic and prognostic biomarker in oral cancer (24). Disease-free survival was analyzed on head and neck cancer samples from The Cancer Genome Atlas (TCGA), and the results revealed that patients with higher MMP1 expression exhibited significantly poorer survival than those with lower MMP1 expression (p = 0.017) (**Figure 2E**). Furthermore, the expression of MMP1, FBLN5, COL5A3 and BGN was significantly increased in patients with perineural invasion (**Figure 2F** **).** Collectively, these results indicate that OSCC patients with high MMP1 expression and/or perineural invasion may benefit from the establishment of their matched PDX models.

### RNA-Seq Analysis Reveals Enriched HIF and PI3K-AKT Pathways in Faster Growing Tumors Compared With Slower Growing Tumors

**Figure 3:**
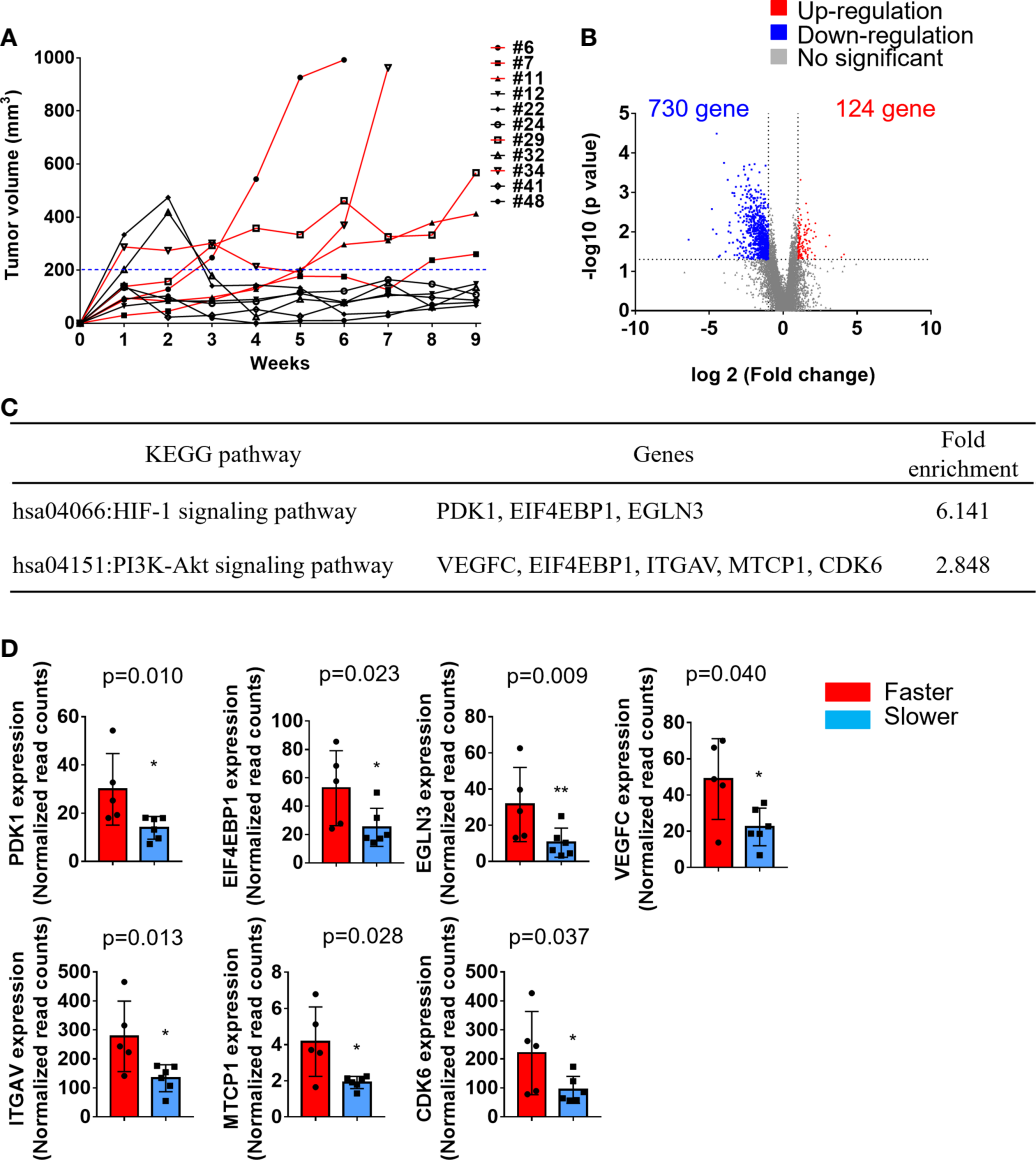
Transcriptomic analysis of faster growing tumors compared with slower growing tumors. Primary tumors were excised and transplanted into immune-deficient mice. The tumor volumes in the flanks of mice were monitored twice a week. **(A)** Tumor growth curve of these 11 PDXs. The patients were divided into either a faster (red lines, tumor > 200 mm^3^) or slower (black lines, tumor < 200 mm^3^) growing group. **(B)** Volcano plot displays the DEGs between faster and slower growing tumors from RNA-seq analysis. The x-axis shows the log2-fold change values, and the y-axis shows the -log10 p values for the differentially expressed genes. **(C)** A total of 124 upregulated and 730 downregulated genes were subjected to KEGG pathway analysis. The significantly upregulated pathways are shown. **(D)** The expression of PDK1, EIF4EBP1, EGLN3, VEGFC, ITGAV, MTCP1, and CDK6 was significantly upregulated in the faster growing group. The p values were calculated using the Mann–Whitney U test. The P value < 0.05 indicated statistical significance. (*p<0.05, **p<0.01).

We also observed that the tumor growth rates were different in these 12 PDX lines. According to the tumor sizes in the first generation within 9 weeks of transplantation in NSG mice, the patients were divided into either faster (tumor > 200 mm^3^) or slower (tumor < 200 mm^3^) growing groups (**Figure 3A**). The transcriptional profiles of the faster (n=5) and slower (n=6) growing groups were compared by RNA-seq analysis of the primary tumors. Compared with the slower growing group, 854 transcripts were significantly altered (2-fold difference, p value < 0.05). Among these, 730 (85.5%) were downregulated and 124 (14.5%) were upregulated in the faster growing group (**Figure 3B** **)**. Kyoto Encyclopedia of Genes and Genomes (KEGG) pathway analysis demonstrated that the upregulated genes were involved in the HIF-1 signaling pathway and PI3K-Akt signaling pathway (**Figure 3C**). The read counts of these genes (PDK1, EIF4EBP1, EGLN3, VEGFC, ITGAV, MTCP1 and CDK6), which are involved in the HIF-1 signaling pathway and PI3K-Akt signaling pathway, were significantly higher in the faster growing group than in the slower growing group (**Figure 3D**). These results may demonstrate that primary tumors under hypoxic conditions or with increased PI3K-Akt activation enhance tumor cell growth in xenografts.

### Genomic Landscapes Are Conserved in Paired Primary Tumors and Xenografts

**Figure 4:**
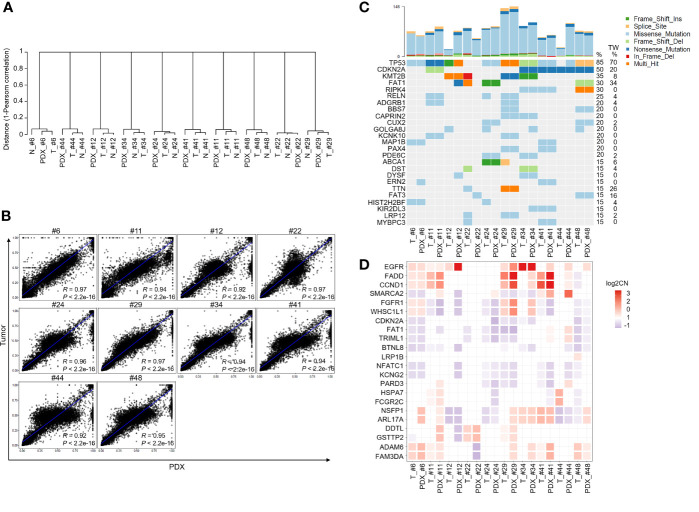
Comparison of genomic landscape alterations in OSCC patients and PDXs. The genomic landscapes of paired normal tissues, tumor tissues, and xenografts were determined by whole-exome sequencing. **(A)** Unsupervised clustering analysis of variant allele frequency (VAF) distributions in paired normal tissues, tumor tissues, and their matched P1 PDXs. **(B)** The correlation coefficients of variants between primary tumors and matched PDXs were calculated. **(C)** The comparison of total genomic mutation counts in OSCC patients and their matched PDXs was shown in the upper panel. The y-axis shows the number of mutation events in the WES data. Heatmap representation of genes frequently mutated between OSCC patients and their matched PDXs. The numbers in the left lane represent the mutation frequencies of specific genes in these 10 paired specimens and in our previously published OSCC cohort (TW; n = 50). **(D)** Heatmap representation of the copy number variation (CNV) of targeted genes in OSCC patients and their matched PDXs.

Next, we determined the DNA mutations and copy number variations (CNVs) in primary tumors and their matched PDXs using WES. Among the 12 PDXs, the quality of genomic DNA from the tumor tissues of 2 patients (patients #7 and #32) was deemed unsuitable for WES. A total of 30 samples, including 10 case-matched patient adjacent normal tissues and tumor tissues and their P1 PDXs, were subjected to WES and analyzed by bioinformatics. Clustering analysis of variant allele frequency (VAF) distributions showed that the matched samples were corrected for paired normal tissues, tumor tissues, and their matched P1 PDXs (**Figure 4A**). The correlation coefficient between primary tumors and their matched PDXs was high and ranged from 0.92 to 0.97 (**Figure 4B**). The top 25 most frequent mutations were also retained in 10 patients with OSCC, and their matched PDXs were listed in **Figure 4C**. Consistent with our previous report, the common mutations in this cohort of 49 OSCC patients were similar to those in most cohorts of Taiwanese OSCC patients (25). In this cohort, these mutations include those in TP53 (n = 8), CDKN2A (n = 5), KMT2B (n = 3), FAT (n = 3), and RIPK4 (n=3) (**Figure 4C**). The numbers of transition and transversion mutations were similar between patients and their matched PDXs (**Supplementary Table 2**).

In addition, WES analysis indicated that the CNVs in individual patients were comparable to those in their corresponding PDXs, including those in significantly amplified/deleted regions encompassing genes such as EGFR, FADD, CCND1, CDKN2A, and FAT1 (**Figure 4D**). Overall, consistent with our previous report, our PDX cohort retained the significantly mutated genes and CNVs from the OSCC Taiwan and head and neck squamous cell carcinoma (HNSCC) TCGA cohorts.

### PDX Models Retain the Gene Expression Profiles of Their Paired Primary Tumors

**Figure 5:**
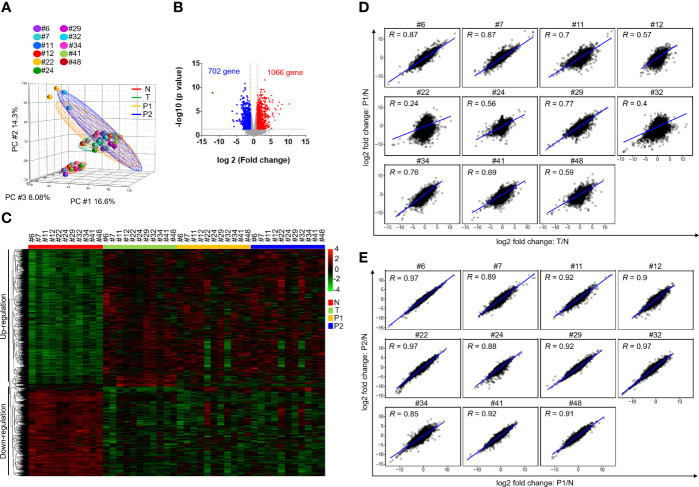
Comparison of transcriptome profiles in OSCC patients and PDXs. The transcriptome profiles of paired normal tissues, tumor tissues, and xenografts were determined by RNA-seq. **(A)** Principal component analysis of the adjacent normal tissues (N), tumor tissues (T) and their matched PDXs at passage 1 (P1) and passage 2 (P2) of OSCC patients. **(B)** Volcano plot of differentially expressed genes between normal tissues and tumor tissues. The x-axis shows the log2-fold change values, and the y-axis shows the -log10 p values for the differentially expressed genes. **(C)** Expression heatmap analysis of transcriptome datasets from the adjacent normal tissues (N), tumor tissues (T) and their matched PDXs at passage 1 (P1) and passage 2 (P2) of OSCC patients. **(D)** Gene expression in patient tumor tissues or PDXs was normalized to that in patient adjacent normal tissues. Correlation of gene expression between patient primary tumor tissues and P1 PDXs. **(E)** Correlation of gene expression between P1 and P2 PDXs.

We then analyzed the transcriptome profiles of these patients and their matched PDXs in the P1 and P2 generations. Of these 12 PDXs, the RNA quality from 1 sample was unsuitable for RNA-seq experiments. A total of 44 case-matched samples, including OSCC patient normal tissues (n = 11), tumor tissues (n = 11), P1 PDXs (n = 11), and P2 PDXs (n = 11), were subjected to RNA-seq and bioinformatics analyses. Our RNA-seq analyses revealed 32,200 genes, and the mean read count was 36.5 ± 0.76 million in the 44 case-matched samples. Principal component analysis (PCA) revealed similar gene expression patterns for case-matched tumors and PDXs (**Figure 5A**). The gene expression profiles of 2 patients (#22 and #32) were slightly different from those of the other 9 patients (**Figure 5A**). Comparative analysis of this transcriptome dataset allowed us to identify 1,768 genes as DEGs (2-fold difference, p value < 0.05, FDR < 0.05); these included 1,066 upregulated and 702 downregulated genes in tumor tissue compared to normal tissue in 11 OSCC patients (**Figure 5B**). Then, the selected DEGs were subjected to clustering analysis to evaluate the dysregulated gene expression profiles of paired primary tumors and xenografts. The hierarchical clustering analysis of 11 case-matched normal tissues, tumor tissues, P1 PDXs, and P2 PDXs revealed clearly separated gene expression profiles between tumor and normal tissue and that PDXs retained the majority of the molecular signatures of their matched patient tissues (**Figure 5C**). The scatterplots displayed the correlation between the PDX RNA-seq and tumor RNA-seq results (**Figures 5D, E**). The Pearson correlation coefficient between matched tumors and PDXs in the P1 generation ranged from 0.24 to 0.87, with a median of 0.7 (**Figure 5D**). We found that the Pearson correlation coefficient between PDX P1 and P2 generation was positively associated, ranging from 0.85 to 0.97 (**Figure 5E**).

**Figure 6:**
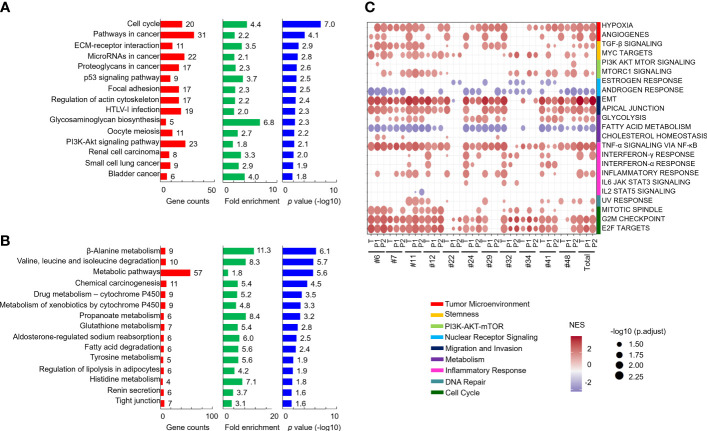
Comparison of the similar pathways in patients and their PDXs. Genes with a 2-fold change and p < 0.05 in both patient tumor tissues and PDXs compared with patient adjacent normal tissues were subjected to KEGG pathway analysis. The results of the pathway analysis are summarized by bar charts and show the gene counts, fold enrichment, and -log p value. The pathways are labeled along the y-axis. The upregulated **(A)** and downregulated **(B)** genes were subjected to pathway annotation. Enriched cancer hallmarks in paired OSCC patients and xenografts were shown in **(C)**.

To explore the dysregulated pathways in patients and xenografts, the 2-fold-upregulated genes with a p value lower than 0.5 in patients or P1 PDXs compared with normal tissues were separately subjected to pathway annotation with DAVID software. KEGG pathway analysis revealed that dysregulated pathways in xenografts were similar to those in patients, such as the ECM-receptor interaction, cell cycle, focal adhesion, and PI3K-AKT signaling pathways (**Supplementary Figure 4**). In addition, compared with normal tissues, the genes with a 2-fold change in tumor tissues, P1 PDXs, or P2 PDXs were subjected to Venn diagram analysis. A total of 650 upregulated and 537 downregulated genes were conserved in primary tumor tissues, P1 PDXs, and P2 PDXs (**Supplementary Figure 5** **)**. KEGG pathway analysis revealed that the 650 upregulated genes were enriched in cancer progression pathways, including the cell cycle, pathways in cancer, and ECM-receptor interactions (**Figure 6A**), whereas the downregulated genes were enriched in metabolic pathways (**Figure 6B**). Furthermore, pathways associated with cancer progression were investigated and plotted according to their normalized enrichment scores (NESs) for the hallmark pathway gene sets, as shown in **Figure 6C**. These results may indicate that our OSCC cohort used to investigate xenograft establishment represents the phenotypes of oral cancer patients and that the transcriptome profiles of these xenografts are similar to those of the primary tumors.

## Discussion

In this study, we established and comprehensively characterized WES and RNA-seq results in paired primary tumors and their matched OSCC PDXs in a Taiwanese population. Our cohort of 49 patients included those with primary tumors who were only newly diagnosed with treatment-naïve OSCC. Among the 49 OSCC samples, 12 successfully generated PDXs, most of which were derived from the buccal mucosa and tongue. The xenograft rate was approximately 25% in treatment-naïve primary tumors in our study. The 12 OSCC xenografts closely represented their parental tumors both in cancer-associated mutations (such as TP53 mutation, CDKN2A mutation, EGFR amplification, and CCND1 amplification) and transcriptome profiles (enriched in the cell cycle, pathways in cancer, ECM-receptor interaction, and PI3K-Akt pathways). Our study also demonstrated that some cellular pathways (hypoxia, PI3K-Akt, and EMT) were associated with high engraftment ability in OSCC. These pathways also contribute to the aggressiveness of various tumor types including oral cancer. In the present study, we first reported that three cancer-associated biological pathways were essential for successful xenograft transplantation.

Paired normal and tumor tissues from patients and their matched PDX models clustered together, demonstrating the relative similarity and stability of the whole-exome analysis between patients and their matched xenografts. Our study validated the use of these preclinical PDX models for OSCC patients and provided a useful biological and preclinical platform for studying tumor biology and testing anticancer therapies, including small molecule inhibitors, monoclonal antibodies, recombinant proteins, or Chinese herbal medicines.

The current study first unveiled that OSCC patients whose cells successfully generated PDXs had a high frequency of perineural invasion, which is a poor prognostic factor for the OSCC treatment outcome (26). Additionally, there was a trend of slightly lower 4-year OS and DFS rates in grafters than in nongrafters. Oral cancer is a highly heterogeneous cancer and genetic variation of individual tumor cells affect patients’ clinical outcomes. Many clinical parameters or molecules have been reported as prognostic biomarkers for oral cancer. The genomics analysis showed that primary tumor with multiple dysregulated pathways have better engraftment rates in our study. However, the 4-year OS and DFS rates were slightly decreased, but no statistical difference, for grafters than for nongrafters. One possibility is the limited sample size in the small cohort; the other possibility is that a marker panel of specific molecules may be better for predicating cancer patients’ survival in oral cancer.

Hypoxia is observed selectively around large tumor masses because of an inefficient blood supply and oxygen delivery (27, 28). Our study revealed that tumor hypoxia, which was not only the top enriched pathway in faster growing xenografts but was also enriched in successful grafters compared with nongrafters, was enriched according to RNA-seq analysis. Hypoxia promotes cancer progression *via* the hypoxia-inducible factor (HIF)-associated signaling pathway, resulting in the increased expression of many oncogenic proteins (29). Hypoxia also promotes drug resistance, including chemotherapy and some targeted therapies, in cancer cells (27). Some reports indicate that hypoxia is a marker of a poor prognosis and chemoradiotherapy failure in HNSCC (30). One of the hypoxia-inducible genes, MMP1, was the best biomarker (AUC = 0.866) for predicting the ability of a patient to successfully generate matched PDXs in our study. It has been reported by us and others that MMP1 in the saliva is a good biomarker for the screening and diagnosis of OSCC (31–33). Furthermore, salivary MMP-1 may be a useful biomarker that is better than CD44 for OSCC diagnosis in the Taiwanese population (31). In the present study, our results indicated that MMP1 expression was significantly increased in patients with perineural invasion and that hypoxic conditions may increase the expression of MMP-1 in human primary tumors, which might help transplanted tumor cells spread and evade in mice.

One limitation of our study is no external validation cohort. There is currently no public database of primary tumor’s genomics analysis between successful grafters and non-grafters in PDXs platform. Our study generated the first public database of comprehensive genomics information for predicting tumor engraftment ability. The patient-derived-xenograft experiment takes an average of three months for establishing one generation of a mouse strain in oral cancer. We will further establish the validation cohort in the future. Some unexpected findings were noted in the current study. Some primary tumor-specific mutations were missed in their matched PDX. The intratumor heterogeneity and different pieces of resected tissues either for genomic sequencing or for transplantation in mice could have caused this inconsistency. Furthermore, some somatic mutations were undetected in xenografts. This may also be explained by the low sequencing coverage or low mutation frequency. Notably, although some variations in gene expression profiles were observed between primary tumors and their matched P1 PDXs, the correlation coefficient of the transcriptome profile between matched P1 and P2 PDXs was at least 85%. The correlation coefficient of some samples was even higher than 97%. These results may be due to specific cell types of these heterogeneous primary tumors surviving and establishing communities in mice. These cell populations have adapted to the new environment and could passage to the next generation, but indeed, some cell populations may be lost in the first xenograft generation.

## Conclusions

Overall, our study demonstrates, for the first time in the literature, that 12 OSCC PDX models were successfully established and the genomic landscapes of paired primary tumor tissues and xenografts were comprehensively profiled by exome-seq and RNA-seq. Our PDX strains maintained common genetic mutation profiles in OSCC, suggesting that this platform might be quite useful for many anticancer drugs that are now widely used in clinical practice. A panel of five genes (MMP1, FBLN5, COL5A3, BGN and LOXL1) was useful for predication the successful grafters among oral cancer patients.

## Data Availability Statement

The sequencing data was deposited to NCBI SRA and the BioProject accession was PRJNA780253.

## Ethics Statement

This study was approved by the Institutional Review Board at Chang Gung Memorial Hospital, Taiwan (Protocol Nos.: 201800700B0, 102-5685A3). Prior to sample collection, written informed consent was obtained from all participants. The patients/participants provided their written informed consent to participate in this study. All animal experiments were conducted in accordance with the Institutional Animal Care and Use Committee of Chang Gung University (Protocol Nos.: CGU106-114 and CGU107-074).

## Author Contributions

W-CY contributed to the experiments, data analysis, and writing and revising the manuscript. IY-FC performed the bioinformatics analysis and writing the manuscript. K-PC contributed to the study design, clinical sample collection, clinical data analysis, and writing and revising the manuscript. C-NO, C-RL, T-LT, Y-CZ, C-IW, Y-HW, AY, HL, C-CW, Y-SC and J-SY contributed to the experiments and provided technical support. C-YY contributed to the study design, experiments, data analysis, and writing and revising the manuscript. All authors have approved the final version of the work.

## Funding

This work was supported by grants from the Ministry of Science and Technology (MOST) (K-PC, MOST 108-2314-B-182A-108-MY3; and C-YY, MOST 110-2314-B-182-046 and MOST 107-2314-B-182-075-MY3) and Chang Gung Memorial Hospital (K-PC, CMRPG3H0853 and CMRPG3J1252; and C-YY, CORPD1J0103, CMRPD1K0402). The authors thank the “Molecular Medicine Research Center, Chang Gung University” from The Featured Areas Research Center Program within the framework of the Higher Education Sprout Project by the Ministry of Education (MOE) in Taiwan (EMRPD1M0281). NGS experiments and bioinformatics analyses were performed at the Genomics NGS Laboratory Molecular Medicine Research Center, Chang Gung University, Taiwan (EMRPD1M0231).

## Conflict of Interest

The authors declare that the research was conducted in the absence of any commercial or financial relationships that could be construed as a potential conflict of interest.

## Publisher’s Note

All claims expressed in this article are solely those of the authors and do not necessarily represent those of their affiliated organizations, or those of the publisher, the editors and the reviewers. Any product that may be evaluated in this article, or claim that may be made by its manufacturer, is not guaranteed or endorsed by the publisher.

## Abbreviations

OSCC, oral cavity squamous cell carcinomas; PDX, patient-derived xenografts; MMP1, matrix metalloproteinase-1; HNSCC, head and neck squamous cell carcinoma; EMT, epithelial–mesenchymal transition; WES, whole-exome sequencing; RNA-seq, RNA sequencing; OS, overall survival; DFS, disease-free survival.
