# Supplementary material for: Genomic and Molecular Signatures of Successful Patient-Derived Xenografts for Oral Cavity Squamous Cell Carcinoma
Source: Front Oncol. 2022 Apr 4;12:792297. doi: 10.3389/fonc.2022.792297 (PMC9013835; doi:10.3389/fonc.2022.792297)
Supplement: Supplementary file 1 [file DataSheet_1.pdf]

Genomic and molecular signatures of successful patient-derived xenografts for oral cavity squamous cell carcinoma

Wei-Chen Yen[1,2]*, Ian Yi-Feng Chang[2,3,]*, Kai-Ping Chang[1,2,4,]*, Chun-Nan Ouyang[2], , Chiao-Rou Liu[6,7], Ting-Lin Tsai[6,8], Yi-Cheng Zhang[6,8], Chun-I Wang[9], Ya-Hui Wang[10], Alice L. Yu[10,11], Hsuan Liu[2,6,12,13], Chih-Ching Wu[1,2,7], Yu-Sun Chang[1,2,6], Jau-Song Yu[2,6,13,14], Chia-Yu Yang[1,2,6,8,#]

Supplementary materials

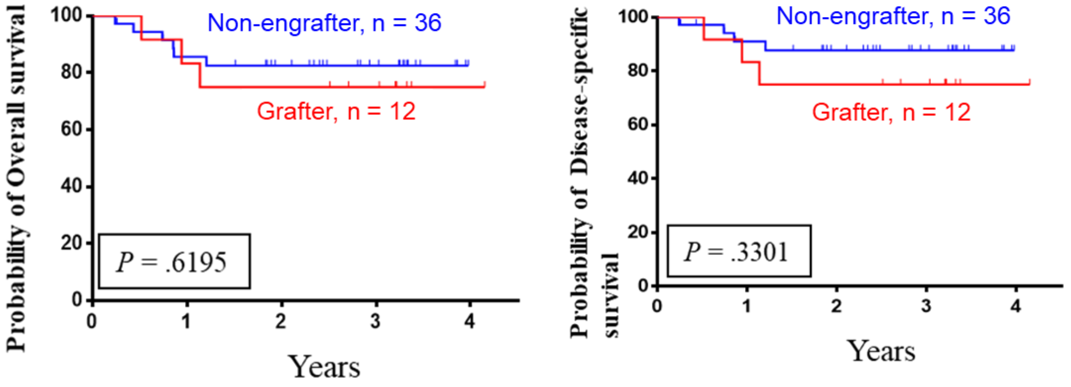

Figure 1. The survival analysis between grafter and non-grafter. Kaplan-Meier plot showing that the 4-year overall survival rates and disease-specific survival rates for patient subgroups stratified by grafter versus non-grafter group. Among the total 49 patients in this cohort, a patient's survival information was not recorded. Survival analysis was conducted for 48 patients.

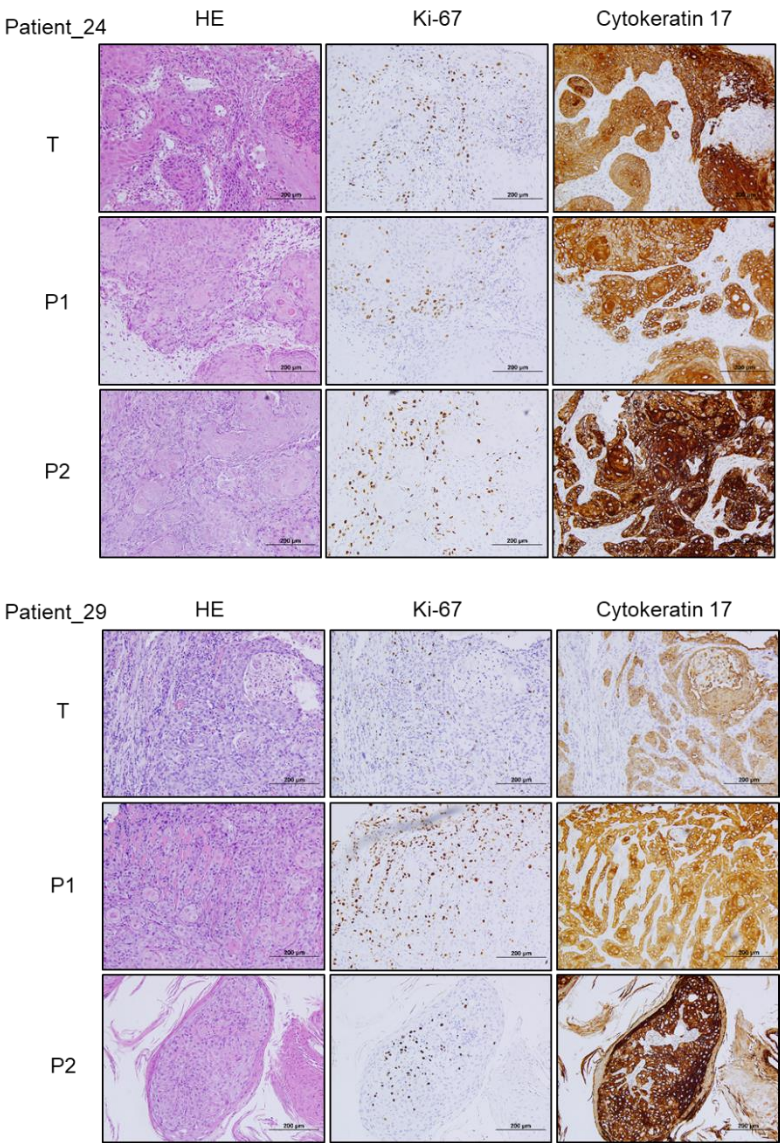

Figure 2. Histological examination of patients and their matched PDX models. Representative histologic images of 2 OSCC patients and their matched PDX tumors. Shown are histology (hematoxylin and eosin staining) comparison between patients' tumors, PDX passage 1 (P1), PDX passage 2 (P2), as well as stain for a cellular marker for proliferation (Ki-67) and cytokeratin 17 for squamous cell carcinoma. Scale bars represent 200 μm. PDXs and corresponding primary tumors revealed the histological similarity.

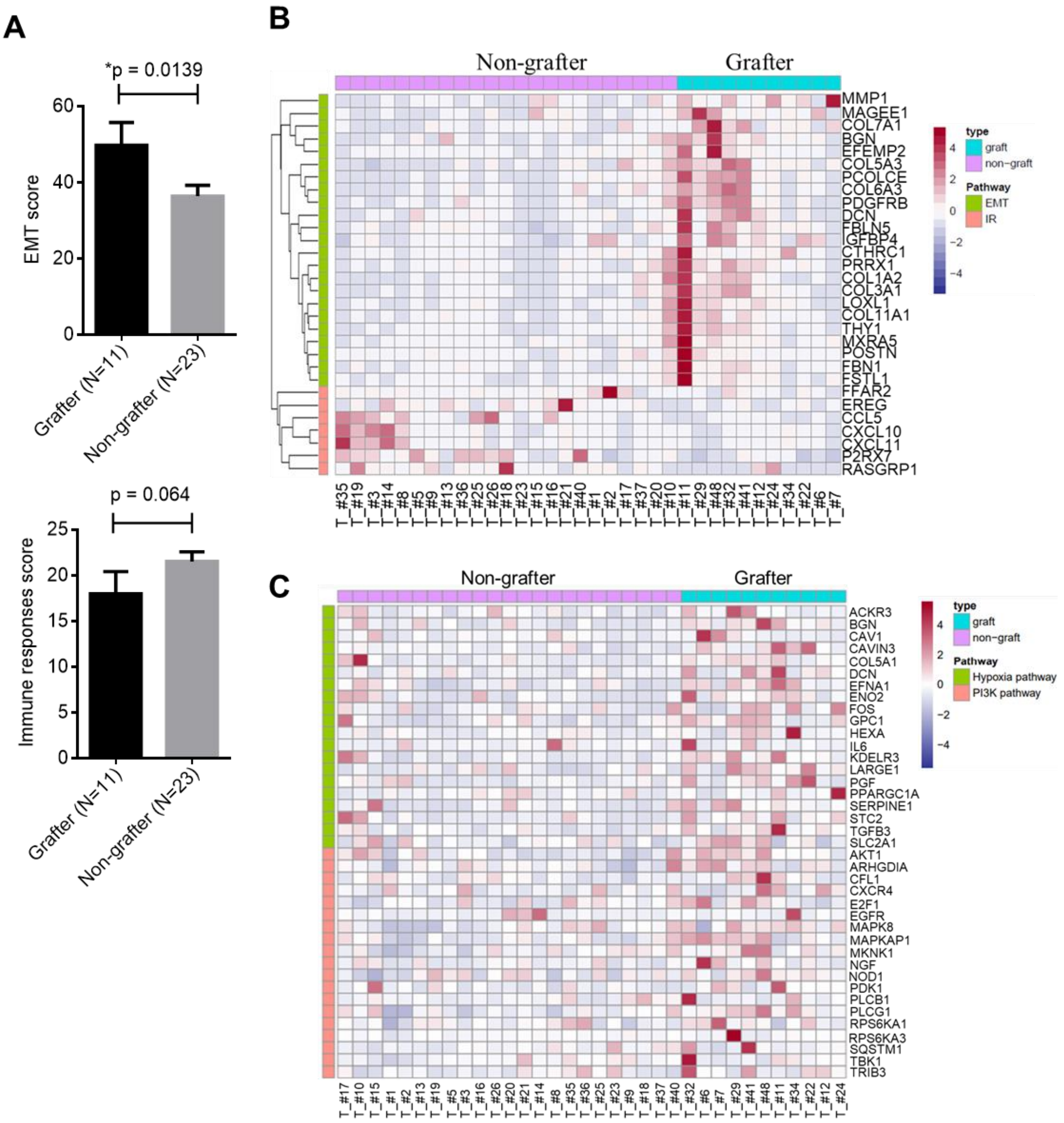

Figure 3. The expression of EMT and immune response-associated genes in OSCC non-grafters and grafters. (A) The genes involved in the regulation of EMT biological process or immune responses were selected and calculated the score based on the geometric mean of the expression levels of genes. The scores of EMT and immune responses between grafters and non-grafters were shown as bar graphs. (B) The heat map of top 30 genes which involved in the regulation of EMT process or immune response (IR) in individual OSCC patients. (C) The heat map of the top differentially

expressed genes involved in hypoxia pathway and PI3K pathway in OSCC patients. The p values were calculated using Mann–Whitney U test. The p value < 0.05 indicated significantly.

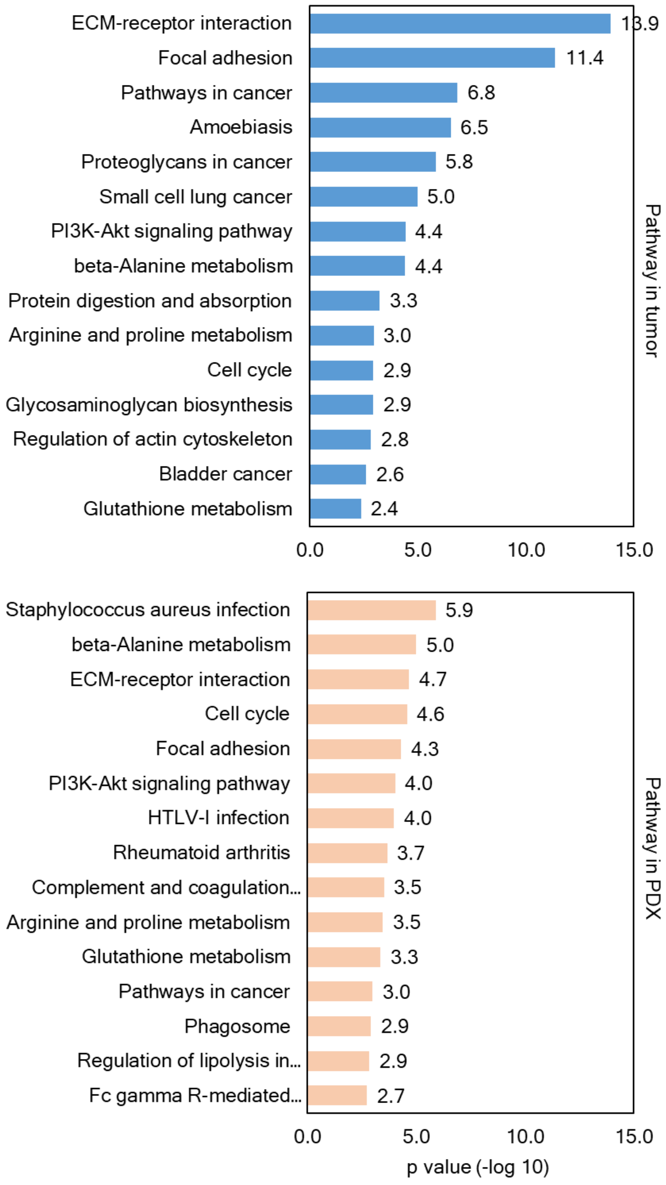

Figure 4. The KEGG pathway analysis of primary tumors and PDXs. Compared with the normal tissues, the dysregulated genes in primary tumors or xenografts were analyzed with the Database for Annotation, Visualization and Integrated Discovery (DAVID) bioinformatics tool (https://david.ncifcrf.gov/). The significantly upregulated pathways in patients (upper panel) and PDX (lower panel) were shown. x-axis represented the –log10 p values. y-axis represented the name of pathways.

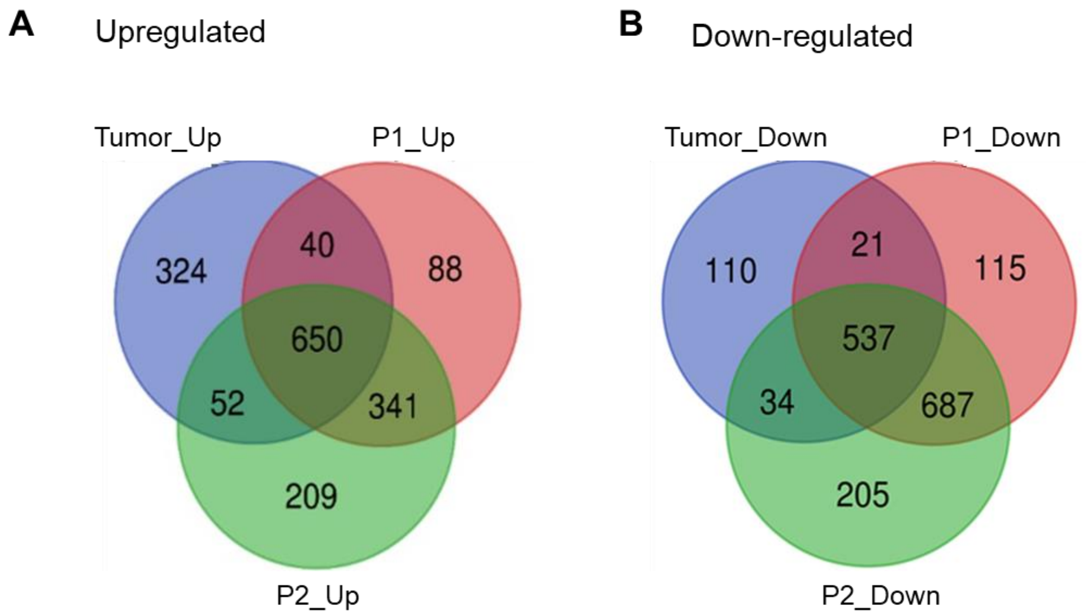

Figure 5. The venn diagram of the differentially expressed genes in primary patients and xenografts. RNA-seq was performed on paired OSCC tumor samples, adjacent normal tissues, and xenografts. Upon read alignment, gene expression levels were determined based on the normalized read count values. Compared with the adjacent normal tissues, the upregulated (A) or downregulated (B) genes (2-fold change, $p < 0.05$) in tumors, P1, and P2 were subjected to venn diagram.

Table 1. The clinical characteristics of oral cancer patients in this study.

Characteristics	Total Specimens	Xenograft		p-value
		No	Yes	
**Number of patients**	49	37	12	
**Pathology**				
**Well**	8 (16.3%)	6 (16.2%)	2 (16.7%)	0.9444
**Moderately**	31 (63.3%)	23 (62.2%)	8 (66.6%)	
**Poorly**	9 (18.4%)	7 (18.9%)	2 (16.7%)	
**NA**	1 (2.0%)	1 (2.7%)	0 (0%)	
**Alcohol drinking**				
**NO**	10 (20.4%)	7 (18.9%)	3 (25%)	0.6497
**YES**	39 (79.6%)	30 (81.1%)	9 (75%)	
**Betel quid chewing**				
**NO**	8 (16.3%)	6 (16.2%)	2 (16.7%)	0.9707
**YES**	41 (83.7%)	31 (83.8%)	10 (83.3%)	
**Cigarette smoking**				
**NO**	8 (16.3%)	5 (13.5%)	3 (25%)	0.3495
**YES**	41 (83.7%)	32 (86.5%)	9 (75%)	
**Site**				
**Buccal mucosa**	19 (38.8%)	14 (37.8%)	5 (41.7%)	0.635
**Tongue**	14 (28.6%)	11 (29.7%)	3 (25%)	
**Gingiva**	3 (6.1%)	3 (8.1%)	0 (0%)	
**Mouth floor**	6 (12.2%)	5 (13.5%)	1 (8.3%)	
**Others**	7 (14.3%)	4 (10.8%)	3 (25%)	

Table 2. The distribution of transition and transversion mutations between patients and their matched PDXs.

Patient number	Transition		Transversion			
	A:T > G:C	C:G > T:A	A:T > C:G	A:T > T:A	C:G > A:T	C:G > G:C
OSCC6_Patient	3	49	35	5	13	5
OSCC6_PDX	16	60	2	3	8	8
OSCC11_Patient	7	44	15	6	12	3
OSCC11_PDX	14	66	4	5	22	7
OSCC12_Patient	2	21	1	1	3	3
OSCC12_PDX	9	60	0	1	14	24
OSCC22_Patient	9	68	2	5	26	6
OSCC22_PDX	13	23	6	5	12	6
OSCC24_Patient	23	40	3	9	21	9
OSCC24_PDX	25	43	4	10	23	12
OSCC29_Patient	20	126	8	5	9	6
OSCC29_PDX	19	126	8	6	12	9
OSCC34_Patient	8	68	7	4	23	12
OSCC34_PDX	9	47	8	2	20	13
OSCC41_Patient	12	45	2	6	3	5
OSCC41_PDX	10	46	3	6	10	5
OSCC44_Patient	6	14	0	1	6	6
OSCC44_PDX	17	69	4	5	19	20
OSCC48_Patient	15	46	1	9	17	21
OSCC48_PDX	11	47	1	6	17	17
